# Beyond the Brain: GABAergic Signaling as a Peripheral Modulator of Skin Barrier Immunity

**DOI:** 10.3390/ijms27156944

**Published:** 2026-08-02

**Authors:** Ruoyu Wan, Wei Hua

**Affiliations:** 1 Department of Dermatology, West China Hospital, Sichuan University, Chengdu 610041, China; wan.ruoyu@outlook.com; 2 Department of Dermatology, West China Hospital Tibet Hospital, Sichuan University, Lhasa 850000, China; 3 Cosmetic Evaluation Center of West China Hospital, Sichuan University, Chengdu 610041, China

**Keywords:** GABA-A receptor, skin barrier, keratinocytes, neuroimmune signaling, calcium homeostasis, ion homeostasis, peripheral neurotransmission, psychodermatology

## Abstract

Beyond its canonical role as the principal inhibitory neurotransmitter in the central nervous system (CNS), peripheral GABAergic signaling has emerged as an important regulator of skin barrier immunity and tissue homeostasis. This review synthesizes current evidence on the expression, distribution, and function of both GABA-A and GABA-B receptors in keratinocytes, fibroblasts, melanocytes, sensory neurons, and cutaneous immune cells, including Langerhans cells, macrophages, mast cells, and T lymphocytes. Particular emphasis is placed on how GABAergic signaling regulates ion transport, calcium dynamics, epidermal differentiation, and immune homeostasis through coordinated interactions with ion channels within the broader cutaneous neuro-immuno-endocrine network. We further discuss evidence supporting the role of cutaneous GABAergic signaling in maintaining barrier integrity, modulating inflammatory responses, and contributing to skin–brain communication within the broader cutaneous neuro-immuno-endocrine network under physiological and pathological conditions. Finally, we summarize the therapeutic potential of targeting peripheral GABAergic pathways in atopic dermatitis, psoriasis, chronic pruritus, photoaging, and other stress-associated dermatoses, highlighting their promise as novel therapeutic strategies in precision dermatology.

## 1. Introduction

The skin is the body’s largest organ and serves as the primary interface between the body and the external environment [1,2]. Beyond its classical role as a physical barrier, the skin is now recognized as an active neuro-immuno-endocrine organ capable of sensing environmental changes and coordinating both local and systemic homeostatic responses [3]. Through complex interactions among epidermal and dermal cells, sensory nerve endings, immune populations, and endocrine signaling networks, the skin integrates diverse environmental cues, including ultraviolet radiation, temperature changes, mechanical stimuli, microbial exposure, and psychological stress [3,4]. These interconnected regulatory systems enable the skin to maintain barrier integrity, immune balance, and tissue adaptation under continuously changing conditions.

Over the past few decades, the concept of cutaneous neuro-immuno-endocrinology has evolved through extensive studies demonstrating that the skin possesses an intrinsic regulatory network involving neurotransmitters, neuropeptides, hormones, cytokines, and growth factors [3,5,6]. Rather than functioning merely as a passive target of systemic signals, the skin can locally produce, sense, and respond to diverse neuroendocrine mediators, enabling bidirectional communication among epithelial, neural, immune, and endocrine compartments [3,4]. Classical examples include neuropeptide signaling mediated by substance P- and calcitonin gene-related peptide (CGRP) [7,8]; neuroendocrine pathways involving corticotropin-releasing hormone (CRH), proopiomelanocortin (POMC), and cutaneous equivalents of the hypothalamic–pituitary–adrenal axis [9,10,11]; and sensory mechanisms mediated by transient receptor potential (TRP) and mechanosensitive ion channels [12,13].

Within the cutaneous neuro-immuno-endocrine network, ion channels represent important molecular interfaces that translate environmental stimuli into cellular responses. By regulating transepithelial ion gradients, pH balance, membrane excitability, and electrochemical homeostasis, ion channels contribute to keratinocyte differentiation, barrier maintenance, tissue repair, and immune regulation [14]. Among these channels, TRP and mechanosensitive ion channels have been increasingly recognized as important mediators of cutaneous environmental sensing and adaptation. Together, these pathways establish the conceptual framework of cutaneous neuro-immuno-endocrine regulation, within which peripheral GABAergic signaling represents an emerging regulatory component.

Beyond classical sensory channels, neurotransmitter-based signaling represents another important layer of regulation within this network. γ-Aminobutyric acid (GABA), the principal inhibitory neurotransmitter in the central nervous system (CNS), was traditionally considered to function primarily within neuronal circuits [15]. However, accumulating evidence has revealed the expression of GABA receptors, metabolizing enzymes, and transporters in peripheral tissues, including the skin, suggesting previously underappreciated roles in barrier regulation, immune modulation, and stress adaptation.

Compared with classical neuropeptide pathways, particularly substance P- and CGRP-mediated signaling, which have been extensively characterized in cutaneous sensory signaling, neurogenic inflammation, vascular responses, and immune regulation, peripheral GABAergic signaling remains comparatively underexplored in dermatological research [16,17]. Understanding the peripheral functions of GABA receptors—particularly GABA-A and GABA-B receptors together with other components of the peripheral GABAergic machinery—in the epidermis and dermis could unveil novel mechanisms for skin homeostasis and potential therapeutic avenues for inflammatory and barrier-disrupted skin diseases [18,19,20]. Emerging evidence further suggests that cutaneous GABA originates from both peripheral sensory nerve endings and local synthesis by resident skin cells, establishing an intrinsic GABAergic network within the skin.

**Figure 1:**
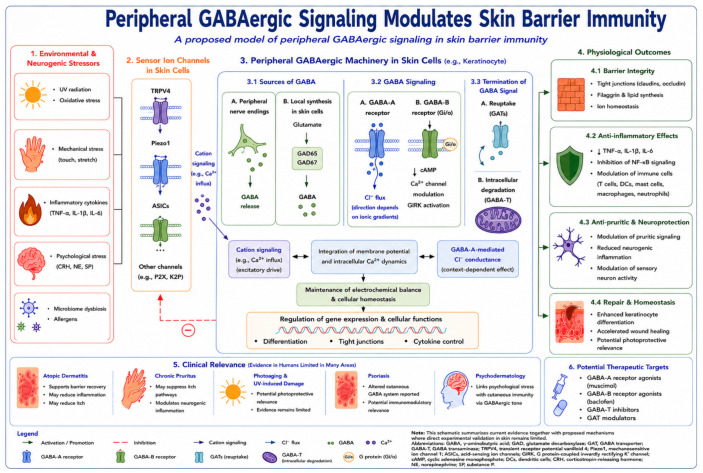
Overview of peripheral GABAergic signaling in skin barrier immunity. Environmental and neurogenic stressors, including ultraviolet radiation, mechanical stimulation, inflammatory cytokines, psychological stress, microbiome dysbiosis, and allergens, activate sensor ion channels such as TRPV4, Piezo1, and ASICs, resulting in intracellular calcium influx. GABA is derived from both peripheral nerve endings and local synthesis within skin cells through glutamate decarboxylases (GAD65 and GAD67). Subsequently, GABA activates both GABA-A and GABA-B receptors, whereas GABA transporters (GATs) and GABA transaminase (GABA-T) terminate signaling through reuptake and degradation, respectively. Together, these pathways form a proposed regulatory network integrating chloride conductance, membrane potential, intracellular calcium dynamics, and downstream cellular responses. Consequently, peripheral GABAergic signaling regulates keratinocyte differentiation, tight junction formation, cytokine production, and barrier homeostasis while influencing fibroblasts, melanocytes, sensory neurons, and cutaneous immune cells. These integrated mechanisms contribute to barrier integrity, inflammatory regulation, neuroprotection, tissue repair, and represent potential therapeutic targets for inflammatory dermatoses, chronic pruritus, photoaging, and psychodermatological disorders. This schematic illustration was developed with the assistance of an AI-based image generation tool and subsequently refined, edited, and verified by the authors for scientific accuracy.

This review aims to provide a comprehensive overview of peripheral GABAergic signaling in skin biology by integrating evidence on GABA-synthesizing and -metabolizing enzymes, GABA transporters, ionotropic and metabotropic GABA receptors, their physiological roles, and their implications in cutaneous pathology. We further emphasize the interactions between GABAergic mechanisms and other epidermal ion channels, highlighting a broader neuro-immuno-endocrine regulatory framework relevant to both physiological homeostasis and cutaneous disease. An overview of these interconnected mechanisms is presented in Figure 1.

## 2. Core Components of Peripheral GABAergic Signaling

Although the GABA system was originally believed to function exclusively within the CNS, accumulating evidence reveals the widespread presence of GABA-synthesizing enzymes, receptors, and transporters in peripheral tissues, including the pancreas, gastrointestinal tract, immune system, and skin [21,22]. These findings have prompted a re-evaluation of GABA as a local paracrine and autocrine signaling molecule beyond its classical neurotransmitter role.

### 2.1. Sources, Transport, and Metabolism of GABA

GABA is synthesized from glutamate by glutamate decarboxylase (GAD), with two primary isoforms—GAD65 and GAD67—responsible for its production [23]. Among the GAD isoforms identified in cutaneous cells, GAD67 has been detected in dermal fibroblasts, supporting an intrinsic capacity for local GABA synthesis within the skin [24]. In addition to local synthesis, cutaneous GABA may also be supplied by peripheral nerve terminals, supporting a complex local GABAergic signaling network within the skin. Betaine/GABA transporter 1 (BGT1), encoded by *SLC6A12*, transports both GABA and betaine and contributes to cellular osmotic adaptation. Given the established importance of organic osmolyte regulation in keratinocyte homeostasis and epidermal barrier function [25,26], BGT1 may be relevant to cutaneous physiology; however, its expression pattern and specific role in cutaneous GABAergic signaling remain poorly characterized. Following receptor activation, GABA signaling is terminated by reuptake through GABA transporters (GATs) and enzymatic degradation by GABA transaminase (GABA-T) [27]. Although these processes have been extensively studied in neurons, the presence and functional relevance of GATs and GABA-T in non-neuronal tissues have also been reported [28].

### 2.2. GABA Receptor Diversity

GABA exerts its physiological effects through three main receptor types, each characterized by distinct structural and signaling properties:

GABA-A receptors are ionotropic, ligand-gated chloride channels that mediate rapid inhibitory transmission. Classical GABA-A receptors are pentameric complexes assembled from combinations of α (1–6), β (1–3), γ (1–3), δ, ε, θ, and π subunits, giving rise to diverse pharmacological profiles, subcellular localization, and kinetic behaviors (phasic vs. tonic activation) [15].

GABA-B receptors are G protein-coupled receptors (GPCRs) composed of GABA-B1 and GABA-B2 subunits. Upon activation, they couple to Gi/o proteins, inhibit adenylyl cyclase, and modulate downstream effectors including voltage-gated calcium channels and G protein-gated inwardly rectifying potassium (GIRK) channels, thereby producing slower and more sustained cellular responses. GABA-B receptors are expressed in both the CNS and peripheral tissues [29].

The broader GABA-A receptor family also includes ρ subunits. Receptors composed predominantly of ρ subunits were historically termed GABA-C receptors but are now classified as GABA-A ρ receptors. These receptors are ligand-gated chloride channels characterized by high GABA affinity, slow desensitization, and distinct pharmacological properties. Although predominantly expressed in the retina, their presence and potential functions in peripheral tissues remain under investigation [30].

### 2.3. Cell Type-Specific Peripheral GABAergic Signaling in the Skin

Current evidence indicates that multiple cutaneous cell types—including keratinocytes, dermal fibroblasts, melanoma cells, and immune cells—harbor components of the peripheral GABAergic machinery (Table 1), highlighting the cell type-specific organization of GABAergic signaling in the skin. Keratinocytes and dermal fibroblasts have been shown to express distinct components of the peripheral GABAergic machinery, supporting the existence of non-neuronal GABAergic signaling in the skin [24,31]. Rather than forming classical synapses, cutaneous GABAergic signaling is thought to operate mainly through local autocrine and paracrine mechanisms [32,33]. In peripheral tissues, GABA-A receptors often exhibit tonic activation, a persistent low-level opening distinct from the transient “phasic” activation observed in synaptic transmission. This tonic activity may play a role in regulating membrane potential and ionic responsiveness in epidermal cells in the absence of synaptic inputs [28,34].

## 3. GABAergic Regulation of Ion Homeostasis in the Skin

### 3.1. Chloride Homeostasis and Membrane Potential

GABA-A receptors are ligand-gated chloride channels whose activation alters chloride conductance and membrane potential according to the prevailing transmembrane chloride gradient [39]. Keratinocytes and dermal fibroblasts co-express GAD67 and functional GABA-A receptors, supporting the existence of a local non-neuronal GABAergic signaling system in the skin [24,40]. In keratinocytes, GABA-A-mediated chloride flux is proposed to regulate membrane potential and contribute to the maintenance of transepithelial ion gradients that are essential for orderly differentiation and epidermal barrier integrity [41,42].

### 3.2. Tonic Versus Phasic Signaling

In neuronal systems, GABA-A receptors operate through two distinct modes of activation: phasic signaling, which is mediated by transient synaptic GABA release, and tonic signaling, which results from persistent activation by low concentrations of ambient extracellular GABA. Because epithelial cells lack classical synaptic structures, tonic activation is likely to represent the predominant mode of peripheral GABAergic signaling.

Evidence from several peripheral non-neuronal cell types supports the functional relevance of tonic or locally sustained GABAergic signaling. In pancreatic α-cells, ambient extracellular GABA tonically activates GABA-A receptors and influences hormone secretion. Likewise, an endogenous autocrine GABAergic signaling system has been identified in the intestinal epithelium, where it regulates epithelial electrolyte transport and fluid secretion through GABA-A receptors [41,43].

### 3.3. Calcium Signaling, Tight Junctions and Barrier Function

Changes in chloride conductance and membrane potential may modulate intracellular Ca^2+^ dynamics, which are important for keratinocyte terminal differentiation and tight-junction assembly [44,45,46]. Exogenous GABA has been shown to increase filaggrin expression in human keratinocytes [47], although the receptor subtype and ionic mechanisms underlying this effect remain to be determined.

Studies using GABA-producing probiotic strains in intestinal epithelial models have reported increased expression of junctional proteins and improved barrier integrity under inflammatory stress [48]. A similar mechanism may operate in the skin, potentially reducing paracellular permeability and transepidermal water loss (TEWL), thereby contributing to epidermal barrier resilience.

## 4. Crosstalk Between GABAergic Signaling and Other Ion Channels

In the skin, ion channels function as environmental sensors, transducing thermal, mechanical, and chemical stimuli into cellular responses that influence barrier integrity, nociception, and inflammation [49]. Among these, TRP channels, Piezo1 mechanosensors, and acid-sensing ion channels (ASICs) play pivotal roles. Although GABA-A receptors are classically inhibitory, emerging evidence suggests that GABAergic signaling may functionally interact with cation-permeable ion channels, including TRPV4, Piezo1, and ASICs, although direct evidence for such interactions in the skin remains limited [50,51].

### 4.1. Electrochemical Coupling with TRP and Piezo1 Channels

TRPV4, a member of the TRP channel family, is expressed in keratinocytes and modulates skin barrier permeability by regulating tight junction proteins such as occludin and claudins [50]. It responds to osmotic stress, heat, and mechanical forces by mediating Ca^2+^ influx, which in turn influences downstream inflammatory pathways. TRPV4-mediated Ca^2+^ influx and GABA-A receptor-mediated chloride conductance exert opposing effects on cellular electrochemical homeostasis. These complementary ionic influences suggest a potential buffering interaction through which GABAergic signaling could modulate TRPV4-driven excitation under inflammatory or osmotic stress conditions. This model remains hypothetical and requires direct electrophysiological validation in keratinocytes [52,53,54].

Piezo1, a mechanosensitive cation channel, governs cellular mechanotransduction in various tissues, including the epidermis. Simultaneous recordings in chondrocytes show that Piezo1 and TRPV4 may operate in parallel or complementary fashion to sense mechanical stimuli [51]. Whether GABAergic signaling directly modulates Piezo1 activity in keratinocytes remains unknown. However, the effects of GABA-A receptors on membrane potential raise the possibility of functional crosstalk with Piezo1-dependent mechanotransduction.

### 4.2. pH and the Ionic Microenvironment

The activity of cutaneous ion channels—including TRP, Piezo1, and ASICs—is strongly modulated by changes in the local ionic microenvironment. For example, ASICs are activated by extracellular acidification and are implicated in inflammation-induced pruritus and skin pain [55]. Although direct evidence is limited, GABA-A-mediated chloride conductance may influence extracellular ion gradients and, indirectly, the gating properties of neighboring cation-permeable ion channels. Additionally, inflammatory acidosis may alter the conformation or activity of GABA-A receptors, potentially affecting their inhibitory tone under stress [56]. These observations suggest that the local pH and the ionic microenvironment may provide a shared regulatory context for multiple ion channel families in the skin.

### 4.3. Integrated Sensory Ion Network

Rather than functioning in isolation, cutaneous ion channels likely form an integrated network that coordinates mechanical, chemical, and immune signals to maintain epithelial homeostasis. TRPV4, Piezo1, and ASICs respond to distinct stimuli yet converge on shared downstream effects such as calcium signaling, barrier remodeling, and cytokine release [57,58]. Within this landscape, GABA-A receptors may provide an inhibitory buffer that counterbalances excitatory signaling, stabilizes membrane potential, and prevents excessive neuroinflammatory responses [56,59]. Decoding this ion channel interplay could reveal novel therapeutic targets for conditions marked by chronic itch, barrier fragility, and neurogenic inflammation.

## 5. Cellular Expression Profiles in Epidermis and Dermis

The skin comprises a diverse population of resident cells—including keratinocytes, fibroblasts, melanocytes, and immune cells—that collectively maintain barrier integrity and coordinate neuro-immuno-endocrine responses. Increasing evidence indicates that these non-neuronal cell populations express distinct components of the peripheral GABAergic machinery (Table 1), supporting cell type-specific roles in local ionic signaling, extracellular matrix remodeling, and immune regulation [24].

### 5.1. Keratinocytes and Fibroblasts in Peripheral GABAergic Signaling

Keratinocytes have been implicated in peripheral sensory transduction and express GABA-A receptor components, though their functional significance in barrier regulation remains under investigation [40].

Dermal fibroblasts express GAD67 and can synthesize GABA, with evidence supporting the GABA-mediated upregulation of elastin and matrix components under oxidative stress [24,36]. These findings suggest that dermal fibroblasts contribute to extracellular matrix remodeling and cutaneous homeostasis through local GABAergic signaling.

### 5.2. Melanocytes and Immune Cells

Melanocytes, situated in the basal layer of the epidermis, have recently been implicated in GABAergic communication with keratinocytes. A study by Tagore et al. demonstrated that B-Raf proto-oncogene, serine/threonine kinase (*BRAF*)-mutated melanoma cells produce and secrete GABA, which binds to GABA-A receptors on neighboring keratinocytes, dampening their electrical activity and promoting melanoma initiation [37]. Although this finding derives from a melanoma model, it provides direct evidence for functional GABAergic communication between melanoma cells and neighboring keratinocytes.

Cutaneous immune cells—including Langerhans cells, dermal dendritic cells, macrophages, and T lymphocytes—also express GABA receptors. GABAergic signaling in immune cells has been shown to inhibit cytokine production, including interleukin-6 (IL-6), interleukin-1 beta (IL-1β), and tumor necrosis factor alpha (TNF-α), and modulate T-cell polarization [60,61,62]. While most studies are not skin-specific, these data support the presence of functional GABA-A receptors in skin-resident immune cells, suggesting that peripheral GABAergic signaling may contribute to local immunoregulation in the skin.

### 5.3. Regulation by Differentiation, UV Exposure, and Stress

Environmental stimuli and cellular differentiation status appear to dynamically regulate cutaneous GABAergic signaling:

- Cellular State and Differentiation: Direct mapping of GABA receptor expression across keratinocyte differentiation states remains lacking. However, the enhanced GABAergic program observed in *BRAF*-mutant melanoma cells and their functional communication with neighboring keratinocytes indicate that cutaneous GABAergic signaling can vary substantially across physiological and pathological cellular states [37].
- Ultraviolet (UV) Radiation: Direct studies on UV modulation of GABA receptors in keratinocytes are scarce. However, given that UV exposure induces oxidative stress and ionic imbalance, it is plausible that GABAergic components respond adaptively under UV-induced stress conditions, though experimental confirmation is required.
- Psychological or Neurogenic Stress: Evidence from broader contexts shows that inflammatory cytokines (e.g., TNF-α, IL-6) can modulate GABA-A receptor function and expression in immune and neuronal networks [60,61,62]. Given the skin’s neuro-immuno-endocrine nature, similar mechanisms may also regulate peripheral GABAergic signaling in the skin.

## 6. Regulation of Keratinocyte Differentiation and Proliferation

Keratinocyte homeostasis depends on a tight balance between basal-layer proliferation and terminal differentiation. GABAergic signaling in these cells—primarily through GABA-A receptors and potentially GABA-B receptors—may regulate proliferation, ion homeostasis, and transcriptional programs associated with epidermal stratification.

### 6.1. GABA-A-Mediated Chloride Conductance and Cell-Cycle Regulation

GABA-A receptors and cyclin D1 regulation: Recent studies in non-cutaneous cell models have shown that knockdown of the GABA-A receptor β3 subunit reduces cyclin D1 expression and induces G1 arrest, suggesting that GABAergic signaling may participate in cell-cycle regulation [63].

GABA-A-mediated chloride conductance and cell-cycle activity: In neural precursor models, GABA-A receptor activation has been shown to alter anion flux, osmotic balance, and cell volume, thereby influencing cell-cycle activity [64].

Ion cross-talk: Changes in chloride conductance and membrane potential may influence calcium dynamics through voltage-sensitive or store-operated calcium-entry pathways, thereby facilitating the expression of differentiation-associated genes.

### 6.2. Chromatin-Mediated Gene Regulation During Differentiation

Chromatin architecture and differentiation: Keratinocyte differentiation involves 3D chromatin remodeling in the Epidermal Differentiation Complex (EDC); enhancer looping precedes the activation of barrier-related genes [65].

GABAergic modulation of epigenetic environment: Because changes in membrane potential and intracellular ion homeostasis have increasingly been recognized as regulators of chromatin organization, GABAergic signaling may influence chromatin remodeling, enhancer activity, and transcription factor accessibility, thereby potentially regulating the expression of differentiation-associated proteins such as involucrin and filaggrin.

### 6.3. Re-Epithelialization and Wound Healing

Beyond homeostatic renewal, GABAergic signaling may contribute to cutaneous repair. Exogenous GABA has been shown to accelerate wound healing in rodent models, partly by promoting fibroblast proliferation, collagen synthesis, and re-epithelialization [47,66]. These effects may involve modulation of membrane potential and downstream calcium influx, thereby facilitating cell migration through ion-dependent mechanisms [67]. In addition, topical application of baclofen, a GABA-B receptor agonist, was reported to reduce epidermal hyperplasia and local inflammation in murine skin, suggesting that GABAergic signaling may contribute to the resolution of inflammation and tissue regeneration [19].

## 7. Barrier Formation, Cytokine Modulation, and Stress Resilience

Beyond its role in regulating keratinocyte proliferation and differentiation, peripheral GABAergic signaling—particularly through GABA-A receptors—has emerged as an important regulator of epidermal barrier function, local immune responses, and stress-associated inflammation. These effects are mediated through the coordinated regulation of intercellular junctions, cytokine production, and neuro-immuno-endocrine communication.

### 7.1. Modulation of Tight Junctions and Barrier Structure

Tight junctions (TJs), composed of proteins such as claudin-1, occludin, and zonula occludens-1 (ZO-1), are essential for maintaining the integrity of the epidermal barrier. The importance of claudin-1 was demonstrated in claudin-1–deficient mice, which exhibited lethal dehydration due to defective skin sealing [68]. Although direct evidence that GABA-A receptor activation regulates epidermal tight junctions remains limited, studies of GABA-producing probiotic strains have reported improved intestinal epithelial barrier integrity and altered junctional protein expression [48]. More broadly, probiotic and epithelial-junction studies demonstrate that the localization and regulation of proteins such as occludin, claudins, and ZO-1 are important determinants of epithelial resilience [69,70].

### 7.2. GABAergic Regulation of Cytokine Production and Immune Activation

Keratinocytes act as immune sentinels and can secrete proinflammatory cytokines like IL-1β, IL-6, and TNF-α in response to external stress or microbial exposure [71]. GABAergic signaling has been shown to suppress the production of these cytokines in both epithelial and immune cells. In models of autoimmune inflammation, GABA treatment significantly inhibited nuclear factor kappa B (NF-κB)-mediated proinflammatory responses [22,72]. This cytokine suppression may contribute to the restoration of epidermal immune homeostasis and interrupt the cycle of chronic inflammation.

### 7.3. Interaction with Stress Pathways and Neuroendocrine Signaling

The skin possesses a local neuroendocrine stress-response system with functional equivalents of the central HPA axis, enabling resident skin cells to produce and respond to CRH, POMC-derived peptides, glucocorticoids, and other stress-related mediators [5,9,10,11]. Peripheral GABAergic signaling may interact with this local stress-response circuitry; however, direct evidence defining such interactions in the skin remains limited. Evidence from broader immunological systems suggests that GABAergic signaling can modulate inflammatory responses under stress-related conditions [60,73].

## 8. Functional Evidence from In Vitro and In Vivo Models

An increasing body of experimental evidence supports the functional role of GABAergic signaling in regulating skin barrier repair, inflammatory responses, and epidermal homeostasis. Both in vitro and in vivo studies have demonstrated the biological activity of GABA receptor agonists in various dermatologic models, highlighting their potential therapeutic relevance.

### 8.1. GABA Receptor Agonists Enhance Barrier Repair and Prevent Hyperplasia

In murine models of barrier disruption, topical application of GABA-A receptor agonists, such as isoguvacine, has been shown to significantly accelerate barrier repair and suppress compensatory epidermal hyperplasia. These effects were reversed by GABA-A receptor antagonists (e.g., bicuculline) but not by GABA-B antagonists, supporting a specific role for GABA-A receptors in epidermal barrier repair [35].

### 8.2. Anti-Inflammatory and Antipruritic Effects in Dermatitis Models

GABAergic agents also exhibit potent anti-inflammatory and antipruritic effects in models of skin inflammation. In a model of allergic contact dermatitis, topical administration of the GABA-B receptor agonist baclofen reduced epidermal thickness, inflammatory cell infiltration, and proinflammatory cytokine production, including TNF-α and IL-1β [18].

Moreover, combined activation of GABA-A and GABA-B receptors produced synergistic inhibition of scratching behavior in an IL-31-driven mouse model of chronic AD-like itch. The long-term enhancement of GABAergic signaling also reduced spontaneous scratching and markedly improved associated skin lesions, supporting complementary GABAergic regulation of pruritic signaling and itch-associated cutaneous pathology [74]. Topical baclofen cream has also demonstrated antiproliferative and anti-inflammatory efficacy in murine skin, further supporting its local therapeutic utility [19].

### 8.3. GABAergic Signaling in Tumorigenesis and Skin Homeostasis

Beyond inflammation, GABAergic signaling may influence tumorigenic processes in the skin. A recent study showed that melanoma initiation is regulated by electrical activity mediated through GABA-A receptors. The study demonstrated that GABA-mediated electrical signaling regulates melanoma initiation through communication between melanoma cells and neighboring keratinocytes [37].

Collectively, these findings provide functional evidence that GABA receptors—particularly GABA-A and GABA-B receptors—are functionally active in the skin. Their pharmacological modulation may represent a promising therapeutic strategy for restoring barrier integrity, limiting cutaneous inflammation, and promoting skin homeostasis.

## 9. GABA Dysregulation in Barrier-Compromised Skin Diseases

Emerging evidence suggests that dysregulation of peripheral GABAergic signaling contributes to the pathophysiology of several barrier-compromised skin diseases, including AD, chronic pruritus, and UV-induced skin inflammation. These conditions are characterized not only by impaired epidermal integrity and immune dysregulation but also by altered neuro-immuno-endocrine communication, processes in which peripheral GABAergic signaling may contribute.

### 9.1. Atopic Dermatitis and Chronic Itch

Several studies have reported altered expression of GABA-A and GABA-B receptor subunits in lesional skin, often associated with reduced GABA synthesis and heightened T helper 2 (Th2)-mediated inflammation. This imbalance between excitatory and inhibitory signaling may contribute to epidermal hyperplasia, sensory nerve hyperresponsiveness, and persistent itch. Preclinical studies using murine models have demonstrated that GABAergic agonists exert both anti-inflammatory and antipruritic effects. Topical baclofen reduced epidermal hyperplasia, inflammatory-cell infiltration, and proinflammatory mediator production in cutaneous inflammation models [18,19]. In parallel, combined activation of GABA-A and GABA-B receptors reduced scratching behavior and improved itch-associated skin lesions in an IL-31-driven chronic itch model [74]. These findings suggest that peripheral GABAergic signaling may exert both anti-inflammatory and antipruritic effects within the skin microenvironment.

### 9.2. UV-Induced Inflammation and Photoaging

In a hairless mouse model, oral GABA administration alleviated several features of UVB-induced skin damage, including erythema, epidermal thickening, reduced hydration, and loss of elasticity [47]. Reconstructed skin models exposed to non-extreme daily ultraviolet radiation exhibit distinct oxidative-stress responses in keratinocytes and fibroblasts [75]. GABA has also been shown to enhance dermal fibroblast survival under oxidative stress [24]. Together, these findings suggest a potential role for peripheral GABAergic signaling in cutaneous stress adaptation, although its precise mechanisms in ultraviolet-induced injury remain to be defined.

### 9.3. Psychological Stress and Peripheral GABAergic Tone

Psychological stress activates cutaneous neuroendocrine mediators, including CRH and catecholamines, and may thereby alter local barrier, immune, and sensory functions [3,4,5,6,9,10,11]. Whether these stress mediators directly modify peripheral GABAergic tone in the skin remains to be determined. GABA-loaded nanoemulsions have shown enhanced skin permeation in formulation studies, supporting the feasibility of topical delivery, although their therapeutic efficacy in stress-related dermatoses has not yet been established [76]. Together, these findings support the therapeutic relevance of modulating peripheral GABAergic signaling within the broader cutaneous neuro-immuno-endocrine communication network in stress-exacerbated inflammatory skin diseases.

## 10. Targeting GABAergic Pathways in Dermatologic Therapy

Increasing recognition of peripheral GABAergic signaling in cutaneous homeostasis and inflammation has spurred interest in developing therapeutic strategies that modulate these pathways. Targeting GABA-A and GABA-B receptors in the skin offers a novel approach to managing inflammatory dermatoses, pruritus, and stress-exacerbated conditions, either as monotherapies or in combination with existing therapies.

### 10.1. Topical and Transdermal Formulations

Current pharmacological approaches targeting peripheral GABAergic signaling in dermatologic research are summarized in Table 2. These interventions include direct GABA-A and GABA-B receptor agonists evaluated in experimental skin models, as well as GABA-related agents investigated for chronic pruritus. Although most evidence remains preclinical, these studies collectively support the therapeutic potential of modulating peripheral GABAergic signaling in inflammatory and barrier-associated skin disorders. Several preclinical and clinical studies have demonstrated the efficacy of pharmacological interventions involving GABA-A and GABA-B receptor agonists, including muscimol and baclofen. These agents have shown antipruritic, anti-inflammatory, and barrier-stabilizing effects in models of AD and contact hypersensitivity [18]. Baclofen cream, for instance, reduced epidermal thickening, mast cell infiltration, and cytokine expression when applied to inflamed skin [18,19].

Although gabapentin and pregabalin do not directly activate GABA receptors, these GABA analogues have shown promise for treating various forms of chronic pruritus. While systemic administration is common in neuropathic itch and postherpetic neuralgia [78,79], recent randomized controlled trials have explored topical gabapentin formulations in patients with uremic pruritus or dialysis-associated itch, with encouraging results [77,80]. Transdermal delivery may offer targeted efficacy with fewer central side effects.

### 10.2. Peripheral GABAergic Signaling in Cutaneous Neuro-Immuno-Endocrine and Skin–Brain Communication

Bidirectional communication between the skin and the central nervous system occurs within a broader cutaneous neuro-immuno-endocrine network [3,4,5,6,9,10,11]. Psychological stress can influence epidermal barrier, immune, vascular, and sensory functions through mediators including CRH, POMC-derived peptides, glucocorticoids, catecholamines, and neuropeptides. Peripheral GABAergic signaling may represent an additional modulatory component of this network; however, whether psychological stress directly alters cutaneous GABAergic tone remains unknown. Accordingly, the therapeutic value of enhancing peripheral GABAergic signaling in stress-exacerbated dermatoses requires direct experimental and clinical validation.

### 10.3. Combination Strategies with Immunomodulators

GABAergic agents may synergize with conventional anti-inflammatory or immunosuppressive therapies, such as topical corticosteroids, calcineurin inhibitors, or Janus kinase (JAK) inhibitors. By regulating membrane potential, reducing cytokine release, and attenuating neurogenic inflammation, GABA-A or GABA-B modulation could enhance treatment responses and reduce required dosages of systemic agents. Combination approaches may be particularly valuable in chronic or refractory skin conditions characterized by both barrier dysfunction and immune dysregulation [18,78].

Overall, current evidence supports the therapeutic potential of targeting peripheral GABAergic signaling in dermatology, especially in light of its immunomodulatory, neurosensory, and stress-buffering properties. Clinical translation will benefit from further pharmacokinetic studies, dermal bioavailability assays, and controlled trials evaluating both efficacy and safety.

## 11. Future Directions and Research Gaps

Although growing evidence supports the involvement of GABAergic pathways in skin barrier regulation, inflammation, and neuro-immuno-endocrine interactions, several unresolved questions continue to limit mechanistic insights and clinical translation. Addressing these gaps will require a concerted, interdisciplinary effort across dermatology, neuroscience, and systems biology.

### 11.1. Cell-Type Specific Roles and Genetic Models

A major challenge lies in delineating the distinct contributions of GABAergic signaling in various cutaneous cell types. While keratinocytes and fibroblasts have been the primary focus of most studies, the roles of melanocytes, immune cells, endothelial cells, and peripheral nerve terminals remain poorly defined. The development and application of cell-specific conditional knockout models, Cre-loxP systems, and lineage-tracing technologies will be crucial to determine the spatial and temporal dynamics of GABA receptor activity in the skin [81].

### 11.2. Advanced Tools to Monitor GABAergic Activity

Direct, real-time visualization of GABAergic signaling in the skin is still lacking. The implementation of chloride-sensitive fluorescent sensors, genetically encoded GABA and pH biosensors, and microfluidic skin-on-chip systems could enable the high-resolution visualization of ion homeostasis, membrane potential changes, and GABA release patterns in response to injury or stress. These tools will be particularly valuable in studying dynamic barrier responses and neuro-immuno-endocrine communication under physiologically relevant conditions.

### 11.3. Multi-Omic and Functional Integration

A comprehensive understanding of peripheral GABAergic function will benefit from integrated transcriptomic, proteomic, and electrophysiological profiling. For instance, single-cell RNA sequencing (scRNA-seq) can help identify subtype-specific expression of GABA receptor subunits across different epidermal and dermal populations [82]. Meanwhile, patch-clamp electrophysiology, calcium imaging, and optogenetic approaches may shed light on how GABA signaling influences skin-cell electrical activity and functional output under both homeostatic and pathological states [83].

### 11.4. Translational Studies and Drug Development

Despite encouraging preclinical findings, a substantial translational gap remains between experimental discoveries and clinical application. Dermal pharmacokinetics, formulation science, and controlled clinical trials are needed to optimize delivery systems, validate therapeutic efficacy, and ensure the safety of GABAergic agents in dermatologic care. In particular, precision dermatology approaches incorporating molecular profiling and patient stratification may enhance treatment responsiveness and reduce systemic side effects.

In sum, future research on peripheral GABAergic pathways in the skin should prioritize cell-type resolution, real-time dynamic analysis, and translational scalability. Bridging these domains will pave the way toward mechanism-based therapies for inflammatory dermatoses, pruritic disorders, and stress-exacerbated skin conditions. Accordingly, peripheral GABAergic signaling should be viewed not as an isolated skin–brain pathway but as an emerging component of the established cutaneous neuro-immuno-endocrine system that integrates epithelial, neural, immune, and endocrine responses.

## Figures and Tables

**Table 1:** Distribution of GABAergic machinery and representative findings across cutaneous cell types.

Cell Type	Peripheral GABAergic Machinery	Representative Findings	Representative References
Keratinocytes	Functional GABA-A-like signaling has been reported, whereas endogenous GABA synthesis, GATs, and GABA-T remain poorly characterized.	GABAergic signaling regulates chloride conductance, epidermal differentiation, barrier recovery, and ion homeostasis.	[35]
Dermal fibroblasts	GAD67-mediated endogenous GABA synthesis. Functional GABA-A receptors have been reported. Transport and degradation pathways remain incompletely defined.	Endogenous GABA regulates extracellular matrix remodeling and elastin synthesis.	[24,36]
Melanoma cells/neighboring keratinocytes	*GAD1*-mediated GABA synthesis in melanoma cells. Functional GABA-A receptor-mediated communication with adjacent keratinocytes.	GABAergic signaling modulates electrical communication and melanoma initiation.	[37]
Macrophages	GABA immunoreactivity and GABA-A receptor expression have been detected.	Increased GABAergic signaling has been reported in psoriatic inflammatory lesions.	[20]
T lymphocytes	GAD67, VGAT, GAT1/2, GABA-T, and multiple GABA-A receptor subunits have been identified.	GABAergic signaling regulates lymphocyte activation, proliferation, and cytokine production.	[38]
Neutrophils	GABA-A receptor immunoreactivity has been reported.	GABAergic components have been detected in inflammatory infiltrates of psoriasis.	[20]

Abbreviations: GABA, γ-aminobutyric acid; GAD, glutamate decarboxylase; GAT, GABA transporter; GABA-T, GABA transaminase; VGAT, vesicular GABA transporter. Note: References shown are representative studies.

**Table 2:** Representative pharmacological interventions targeting GABA-related pathways in dermatologic research.

Agent	Mechanism of Action	Experimental Model/Clinical Indication	Route	Main Finding	Evidence Level	Reference
Isoguvacine	GABA-A receptor agonist	Experimental cutaneous barrier disruption	Topical	Accelerated barrier recovery and reduced compensatory epidermal hyperplasia	Preclinical	[35]
Baclofen	GABA-B receptor agonist	Allergic contact dermatitis and murine skin inflammation	Topical	Reduced epidermal hyperplasia, inflammatory infiltration, and inflammatory mediator production	Preclinical	[18,19]
Muscimol + baclofen	GABA-A and GABA-B receptor agonists	Mouse model of AD-like pruritus	Experimental administration	Produced synergistic inhibition of scratching behavior	Preclinical	[74]
Topical gabapentin	α2δ calcium-channel ligand; not a GABA receptor agonist	Dialysis-associated pruritus	Topical	Reduced pruritus in a randomized controlled study	Clinical	[77]

Note: Gabapentin is included because of its clinical relevance to chronic pruritus and its structural relationship to GABA; however, it does not directly activate GABA receptors and primarily binds the α2δ subunit of voltage-gated calcium channels.

## Data Availability

No new data were created or analyzed in this study. Data sharing is not applicable to this article.
